# High-mobility group box 1 released from astrocytes promotes the proliferation of cultured neural stem/progenitor cells

**DOI:** 10.3892/ijmm.2014.1820

**Published:** 2014-06-24

**Authors:** MAN LI, LIN SUN, YONG LUO, CHENCHEN XIE, YUESHAN PANG, YUAN LI

**Affiliations:** 1 Department of Neurology, The First Affiliated Hospital of Chongqing Medical University, Chongqing 40016, P.R. China; 2 Department of Orthopedics, Shanxi Academy of Medical Sciences, Shanxi Dayi Hospital, Taiyuan, Shanxi 030032, P.R. China; 3 Chongqing Key Laboratory of Neurology, Chongqing 40016, P.R. China; 4 Basic Medicine College of Shanxi Medical University, Taiyuan, Shanxi 030001, P.R. China

**Keywords:** high-mobility group box 1, neural stem/progenitor cell, reactive astrocyte, proliferation, receptor for advanced glycation endproducts, JNK

## Abstract

Astrocytes are major components of the adult neurogenic niche and play a crucial role in regulating neural stem cell proliferation and differentiation. Following brain injury, astrocytes become reactive and release high-mobility group box 1 (HMGB1), which plays a crucial role in the inflammatory process. However, although it has been reported that HMGB1 promotes neural stem/progenitor cell (NS/PC) proliferation in the developing brain, whether HMGB1 released by reactive astrocytes regulates NS/PC proliferation remains unknown. In this study, we aimed to investigate whether HMGB1 released from reactive astrocytes enhances NS/PC proliferation and to elucidate the possible mechanisms involved in this process. To evaluate the effects of HMGB1 on NS/PC proliferation, NS/PCs were cultured in HMGB1 culture medium and astrocyte-conditioned medium with or without reactive astrocyte-derived HMGB1 by RNA interference (RNAi). To explore the possible mechanisms, the HMGB1 receptor for advanced glycation endproducts (RAGE) in the NS/PCs was blocked with anti-RAGE antibody, and c-Jun N-terminal protein kinase (JNK) in the NS/PCs was inhibited using the potent JNK inhibitor, SP600125. Our results suggested that HMGB1 released from reactive astrocytes promoted NS/PC proliferation *in vitro*, and the blockade of RAGE or the inhibition of the JNK signaling pathway in the NS/PCs prevented the HMGB1-induced NS/PC proliferation. Our findings demonstrated that HMGB1 released by reactive astrocytes promoted NS/PC proliferation by binding RAGE and enhancing the phosphorylation of the JNK signaling pathway. These findings support a previously described mechanism of a crosstalk between astrocytes and NS/PCs, and suggest that reactive astrocyte-derived HMGB1 plays an important role in the repair of the central nervous system following brain injury.

## Introduction

Astrocytes are major components of the adult neurogenic niche and play a crucial role in regulating neural stem cell proliferation and differentiation (1–4). Following acute brain injury, such as cerebral ischemia, intracerebral hemorrhage (ICH) or traumatic brain injury, astrocytes become activated and proliferate in the context of brain damage (5–7). Reactive astrocytes secrete numerous pro-inflammatory cytokines which play pathophysiological roles in the subsequent damage.

High-mobility group box 1 (HMGB1) protein is one of the most important pro-inflammatory cytokines secreted by reactive astrocytes (8,9), and mediates a variety of inflammatory responses following insults that include sepsis, pancreatitis and pneumonia (10–12). HMGB1 is a highly conserved non-histone DNA-binding protein that stabilizes nucleosome formation and regulates gene expression by facilitating transcription (13,14). Previous studies have confirmed that following brain injury, HMGB1 acts as an important pro-inflammatory cytokine in mediating inflammation, neuronal apoptosis and tissue damage (15–18). However, emerging data suggest that HMGB1 also appears to have beneficial effects during the recovery stage of brain injury. HMGB1 promotes neurovascular remodeling by recruiting endothelial progenitor cells (EPCs) and increasing EPC proliferation (7,19,20). Roles for HMGB1 in brain development have also been reported, as HMGB1 is associated with neurogenesis, neural progenitor cells survival and proliferation, neurite outgrowth and neuronal differentiation (21–23).

Following brain injury, endogenous neural stem/progenitor cells (NS/PCs) spontaneously proliferate in proximity to the damaged area (24–26). However, the precise mechanisms driving the NS/PC spontaneous proliferation in response to damage remain unknown. Considering that HMGB1 can induce NS/PC proliferation, and is secreted by reactive astrocytes following brain injury, it is highly possible that HMGB1 released by astrocytes may potentiate NS/PC proliferation following brain injury.

In the present study, we examined the hypothesis that HMGB1 released by astrocytes promotes NS/PC proliferation, and investigated the possible intracellular signaling pathways within the NS/PCs involved in this process. Astrocytes were stimulated with low levels of interleukin (IL)-1β to mimic a reactive phenotype. Subsequently, we used astrocyte-conditioned medium (ACM) with or without HMGB1 through the use of RNA interference (RNAi) to evaluate the effects of astrocyte-derived HMGB1 on NS/PC proliferation. We then explored the potential underlying mechanisms *in vitro*, through the blockade of the receptor for advanced glycation endproducts (RAGE) with an anti-RAGE antibody and by inhibiting the c-Jun N-terminal protein kinase (JNK) signaling pathway with the potent JNK inhibitor, SP600125. The present study provides an experimental basis to explain the potential mechanisms through which NS/PCs spontaneously proliferate following brain injury.

## Materials and methods

### Animals

The Ethics Committee of Chongqing Medical University, Chongqing, China approved all protocols for the animal experiments. All procedures were carried out in accordance with the National Institutes of Health (NIH) Guide for the Care and Use of Laboratory Animals (NIH, Bethesda, MD, USA). All animals were provided by the Experimental Animal Center of Chongqing Medical University. Neonatal Sprague-Dawley (SD) rats (1–2 days old) were used for primary astrocyte culture and primary NS/PC culture. All efforts were made to minimize the number of animals used, as well as their suffering.

### Study design

In the experiments using ACM, the astrocytes were assigned to 4 groups, and cultured for 24 h in serum-free NS/PC medium with or without IL-1β (0.1 ng/ml), as previously described by Hayakawa *et al* (7) (Table I). Four types of conditioned medium were collected and used undiluted in NS/PC proliferation assays: normal ACM (nACM), stimulated ACM (sACM), HMGB1 shRNA interference sACM (HMGB1 shRNA sACM) and control shRNA interference sACM (control shRNA sACM).

In the NS/PC proliferation experiments, the NS/PCs were assigned to 6 groups, and cultured in NS/PC culture medium (vehicle), nACM with IL-1β (0.1 ng/ml) (control), sACM, HMGB1 shRNA sACM, control shRNA sACM and HMGB1 culture medium (Table I). To eliminate interference by IL-1β and cytokines that were secreted by normal astrocytes, we used nACM containing IL-1β (0.1 ng/ml) as a control culture (control group). To further confirm that HMGB1 promotes NS/PC proliferation, we added 7 ng/ml recombinant human HMGB1 (approximate to the HMGB1 level in sACM) to serum-free NS/PC medium as an additional control (HMGB1 group). Each group was further divided into 4 subgroups for time course analysis and cultured for 24, 48, 72 and 96 h.

In the analysis of the dual RAGE-JNK signaling pathway, we selected HMGB1 culture medium (NS/PC culture medium containing 7 ng/ml recombinant human HMGB1). In the RAGE pathway experiments, the NS/PCs were cultured in HMGB1 culture medium in the presence of IgG (20 μg/ml) or anti-RAGE antibody (20 μg/ml) for 96 h (Table I). In the JNK signaling pathway experiments, the NS/PCs were cultured in HMGB1 culture medium in the presence of increasing concentrations of the potent JNK inhibitor, SP600125 (0, 1 and 10 μM), and serum-free NS/PC culture medium for 96 h (Table I).

### Primary astrocyte culture

Primary astrocytes were prepared from the cortices of 1–2-day-old neonatal SD rats as previously described (7), with some modifications. The cells were grown in high-glucose Dulbecco’s modified Eagle’s medium (DMEM; Invitrogen, Beijing, China), containing 10% fetal bovine serum (FBS; Gibco, Life Technologies Ltd., Mount Waverley, Victoria, Australia) and 25 μg/ml penicillin/streptomycin in 95% air/5% CO_2_. To remove non-astrocytic cells, which were mainly microglia, the flasks were shaken overnight at 200 rpm. The cells were then trypsinized and seeded into T50 flasks at a density of 6x10^5^ cells/cm^2^. The cells were cultured for 16–18 days before preparing the ACM and were only used for study when the purity of the astrocytes reached a level of 95% [verified with Glial fibrillary acidic protein (GFAP) staining].

### Primary NS/PC culture

NS/PCs were prepared from the cortices of 1–2-day-old neonatal SD rats as previously described (27), with minor modifications. Single cells were plated in uncoated T50 flasks at a density of 1x10^5^ cells/cm^2^ with NS/PC culture medium composed of DMEM/F12 (Invitrogen) supplemented with 2% B27 supplement (Invitrogen), 20 ng/ml epidermal growth factor (EGF; Peprotech, Rocky Hill, NJ, USA) and 20 ng/ml basic fibroblast growth factor (bFGF; Peprotech). The cultures were maintained for 7 days in a humidified incubator at 37°C and 5% CO_2_, with half-volume changes of fresh medium every 2–3 days. Neurospheres that formed during the period were dissociated into single cells with 0.125% trypsin (HyClone, Logan, UT, USA), and the cells were regenerated into neurospheres using NS/PC culture medium. These procedures were repeated 2–3 times before the cells were treated and single-cell suspensions from neurospheres were utilized to examine cell proliferation.

### Lentivirus infection of astrocytes

Lentiviral vectors expressing HMGB1 shRNA or control shRNA were obtained from Shanghai Genechem Co. Ltd, Shanghai, China. The sequences of HMGB1 shRNA were as follows: 5′-GATCCCGAAGCA CCCGGATGCTTCTTTCAAGAGAAGAAGCATCCGGG TGCTTTTTTGGAAA-3′ (15). Control shRNA consisted of a scrambled sequence that fails to target any known cellular mRNA.

The shRNA-carrying lentiviral vector was used to infect the astrocytes at a multiplicity of infection (MOI) of 10, 30 and 50. The lentiviral vectors were prepared according to the transduction protocol for cell cultures provided by Shanghai Genechem. Five days after infection, the cells were collected and the percentage of GFP-positive cells was quantified by fluorescence microscopy. The optimum MOI was found to be 30, which resulted in a transduction efficiency of 95%. Western blot analysis was then performed to determine the efficiency of HMGB1 knockdown.

### Enzyme-linked immunosorbent assay (ELISA)

The concentrations of HMGB1 in the nACM, sACM, HMGB1 shRNA sACM and control shRNA sACM were analyzed with a commercially available ELISA kit according to the manufacturer’s instructions (Cat. no. CSB-E08224r/96T; Cusabio Biotech Co., Ltd., Wuhan, China).

### CCK-8 proliferation assays for NS/PCs

The CCK-8 (Cat. no. C0038; Beyotime Institute of Biotechnology, Shanghai, China) proliferation assay was used to determine the rate of NS/PC proliferation. Single NS/PCs were seeded into 96-well plates (10,000 cells/well in 100 μl medium) with corresponding medium. CCK-8 solution (10 μl) was added to the cell culture medium and following incubation for 2 h at 37°C, the absorbance of the culture medium was determined at a wavelength of 450 nm with a reference wavelength of 630 nm by using a multiskan spectrum scanning spectrophotometer (Thermo Labsystems, Vantaa, Finland). Using these procedures, a good linear correlation was obtained between the absorbance and viable cell number. Each experiment was performed in triplicate and the results were collected as the average of at least 3 independent experiments.

### NS/PC cell cycle analysis

The effects of the different culture media on cell cycle distribution were measured by flow cytometry, as previously described (28). The NS/PCs were dissociated into single-cell suspensions, and fixed in 70% ice-cold ethanol overnight at 4°C. The fixed cells were stained with propidium iodide (50 μg/ml; BD Biosciences Franklin Lakes, NJ, USA), and 50 μg/ml RNAse A (BD Biosciences) at 37°C for 30 min in the dark, and subsequently analyzed using a flow cytometer (FACSVantage SE; BD Biosciences). The changes in cell cycle distribution were determined by calculating the proliferation index (PI). The following formula was used: PI = (S + G2/M)/(G0/G1 + S + G2/M), as previously described (28).

### Double-immunofluoresence labeling

The cultured astrocytes and neurospheres were washed and fixed with 4% paraformaldehyde at 4°C for 30 min. The astrocytes were incubated overnight at 4°C with the following primary antibodies: mouse anti-GFAP (1:300; Cell Signaling Technology, Danvers, MA, USA) and rabbit anti-HMGB1 antibodies (1:100; Abcam, Cambridge, MA, USA). Neurospheres were incubated overnight at 4°C with the following primary antibodies: rabbit anti-nestin (1:50; Proteintech, Wuhan, China) and mouse anti-Sox-2 antibodies (1:100; Cell Signaling Technology). The cells were then incubated with secondary antibodies, including goat anti-mouse Alexa Fluor 647 (1:200; Beyotime Institute of Biotechnology), goat anti-rabbit TRITC (1:100) and goat anti-mouse FITC (1:100) (all from Beijing Ding Guo Changsheng Biotech Co., Ltd., Beijing, China) for 1 h at 37°C in the dark. Finally, the cells were examined by laser-scanning confocal microscopy on an Olympus IX70 inverted microscope (Olympus, Tokyo, Japan) equipped with a FluoView FVX confocal scan head (Leica Microsystems GmbH, Wetzlar, Germany).

### Western blot analysis

Total protein derived from the astrocytes and NS/PCs was harvested with RIPA lysis buffer containing a protease and phosphatase inhibitor cocktail (KeyGen Biotech Co., Ltd., Nanjing, China). The protein concentrations were measured using the Bradford method (Beyotime Institute of Biotechnology). The protein samples (50 μg) were fractionated by 10% SDS-polyacrylamide gel electrophoresis, electroblotted onto a polyvinylidene difluoride (PVDF; Millipore, Billerica, MA, USA) membrane and immunoblotted with primary antibodies, including: rabbit anti-HMGB1 (1:1,000), rabbit anti-phosphorylated JNK (anti-p-JNK, 1:1,000; Cell Signaling Technology), rabbit anti-JNK (1:1,000; Cell Signaling Technology) and mouse anti-β-actin anbitody (1:1000, Beijing Ding Guo Changsheng Biotech) as an internal control. A Bio-Rad apparatus (Bio-Rad Laboratories, Richmond, CA, USA) and Quantity One software version 4.6.2 (Bio-Rad Laboratories) were used to scan the immunoblots for semi-quantitative analysis.

### Statistical analysis

The statistical software program SPSS 18.0 for Windows (SPSS Inc., Chicago, IL,USA) was used to conduct statistical analysis. All data are presented as the means ± standard deviation (SD). Statistical analysis was performed by one-way ANOVA, and when α values were at P<0.05, the Bonferroni test was used for homogeneity of variance, and the Tamhane test was used for heterogeneity of variance. Values of P<0.05 were considered to indicate statistically significant differences.

## Results

### IL-1β-stimulated astrocytes can release HMGB1, and RNAi successfully suppresses the release of HMGB1

**Figure 1:**
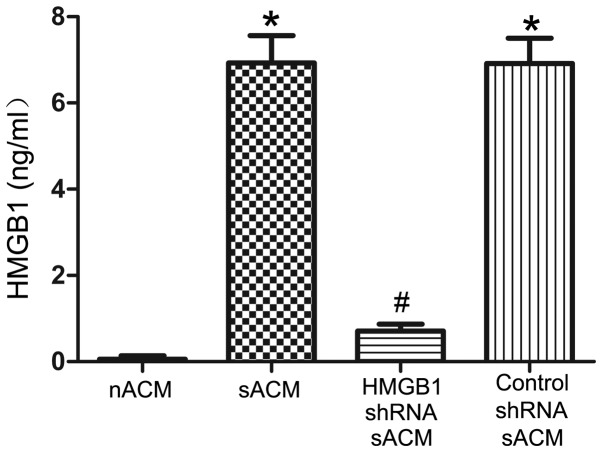
Release of HMGB1 from IL-1β-stimulated astrocytes. ELISA confirmed that HMGB1 was undetectable in normal astrocyte-conditioned medium (nACM). IL-1β (0.1 ng/ml) stimulation caused astrocytes to release HMGB1 into stimulted astrocyte-conditioned medium (sACM). HMGB1 shRNA attenuated the release of HMGB1 into the ACM (HMGB1 shRNA sACM). A control shRNA (control shRNA sACM) had no effect on HMGB1 release into IL-1β-stimulated ACM. ^*^P<0.001 vs. nACM; ^#^P<0.001 vs. sACM.

To determine the effects of IL-1β and RNAi on HMGB1 secretion, we examined the HMGB1 concentrations in the ACM. HMGB1 protein expression was barely detectable in the nACM (0.060±0.078 ng/ml). After 24 h of stimulation with IL-1β, sACM showed a clear accumulation of soluble HMGB1 (6.929±0.630 ng/ml). Compared with that in sACM, the HMGB1 concentration in the HMGB1 shRNA sACM (0.707±0.164 ng/ml) was markedly decreased by RNAi (Fig. 1). Additionally, there was almost no effect on the HMGB1 concentration in the control shRNA sACM (6.917±0.586 ng/ml), compared with that in the sACM (Fig. 1).

### IL-1β upregulates HMGB1 protein expression in astrocytes, and RNAi successfully suppresses the expression of HMGB1

**Figure 2:**
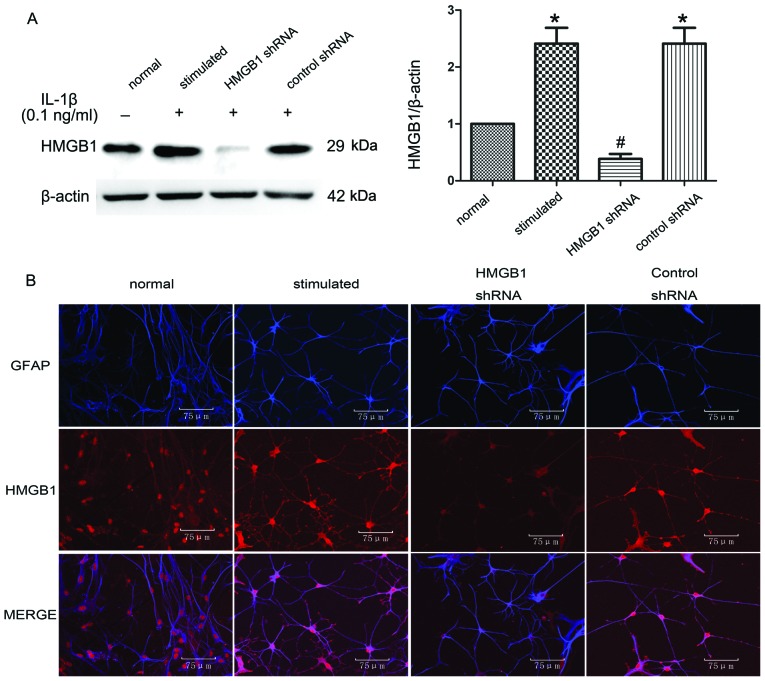
(A) Western blot analysis and (B) double-immunofluorescence labeling of HMGB1 expression in IL-1β-stimulated astrocytes. (A) HMGB1 protein expression was upregulated in IL-1β-stimulated astrocytes. HMGB1 shRNA specifically suppressed HMGB1 protein expression in IL-1β-stimulated astrocytes, as a control shRNA had no effect on HMGB1 protein expression in IL-1β-stimulated astrocytes. ^*^P<0.001 vs. unstimulated astrocytes; ^#^P<0.001 vs. stimulated astrocytes. (B) Double-immunofluorescence labeling of astrocytes for GFAP (blue) and HMGB1 (red). HMGB1 expression was upregulated in IL-1β-stimulated GFAP^+^ astrocytes. HMGB1 shRNA successfully suppressed HMGB1 expression in IL-1β-stimulated GFAP^+^ astrocytes. There was no difference in HMGB1 expression between IL-1β-stimulated GFAP^+^ astrocytes and IL-1β-stimulated control shRNA astrocytes.

To determine the effects of IL-1β and RNAi on intracellular HMGB1 expression, we examined the HMGB1 protein levels in cell lysates obtained from astrocytes and double-immunofluorescence-stained astrocytes. Western blots of astrocyte lysates (Fig. 2A) and the double-immunofluorescence staining of astrocytes (HMGB1/astrocytes red/blue; Fig. 2B) confirmed that endogenous HMGB1 protein was indeed upregulated in the IL-1β-stimulated astrocytes compared to the normal astrocytes. HMGB1 shRNA effectively suppressed HMGB1 protein expression in the IL-1β-stimulated astrocytes. Control shRNA had no effect on HMGB1 expression in the IL-1β-stimulated astrocytes, compared to that of the IL-1β-stimulated astrocytes.

### Neurospheres cultured in vitro were identified with the representative markers, nestin and SRY-re lated HMG-box gene 2 (Sox-2)

**Figure 3:**
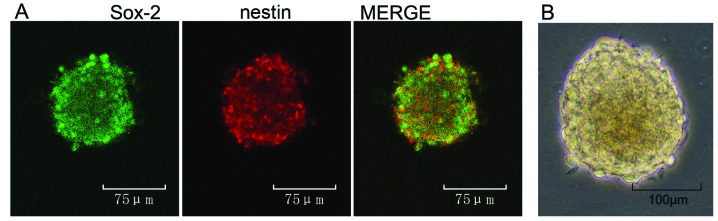
Identification of neurospheres. (A) Neurospheres were identified by the representative markers, Sox-2 (green) and nestin (red). (B) A representative neurosphere observed under a light microscope.

To characterize the cell population, we performed double-labelling experiments with markers of undifferentiated neural stem/progenitor cells, including the intermediate neurofilament protein, nestin, and Sox-2, a transcription factor expressed in undifferentiated stem/progenitor cell nuclei (29) (Fig. 3A). Infant rat cortex-derived neurospheres grown in the presence of EGF and bFGF are mainly composed of nestin (red) and Sox-2 (green)-positive cells. The results of double-immunolabelling experiments and the light microscopy of neurospheres (Fig. 3B) suggested that they are composed of undifferentiated NS/PCs.

### HMGB1 released by astrocytes promotes NS/PC proliferation in vitro

**Figure 4:**
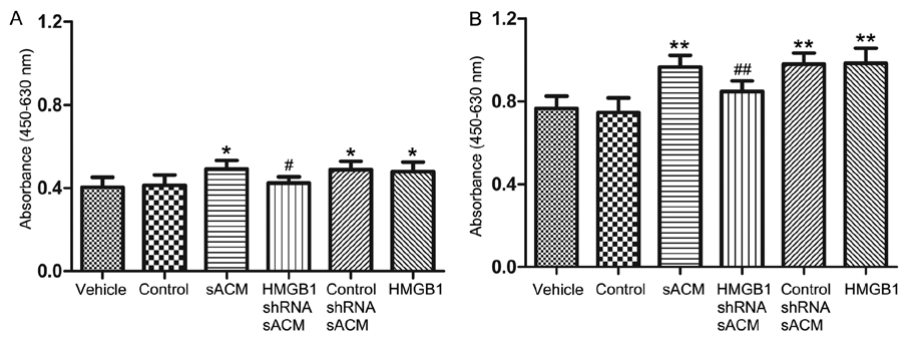
The effects of astrocyte-derived HMGB1 on NS/PC proliferation were measured by the CCK-8 assay. Relative absorbance values of viable NS/PCs were measured by CCK-8 assay at (A) 72 h and (B) 96 h. The proliferation of NS/PCs in sACM, control shRNA sACM and HMGB1 culture media was increased following 72 and 96 h of exposure. Compared with the sACM, the proliferation of NS/PCs in HMGB1 shRNA sACM significantly decreased after 72 and 96 h. There were no intra-time point differences in NS/PC proliferation between the vehicle (NS/PC culture medium only) and control (normal ACM with IL-1β) groups at either the 72- or 96-h timepoint. ^*^P<0.01, ^**^P<0.001 vs. vehicle group; ^#^P<0.05, ^##^P<0.01 vs. sACM group.

To determine whether HMGB1 released from astrocytes affects NS/PC proliferation, we performed an *in vitro* CCK-8 assay. The absorbance of CCK-8 in the NS/PC proliferation experiments is shown in Table II. The results indicatedd that there were no differences in the proliferation rates among each of the groups at 24 or 48 h (P>0.05). However, as shown in Fig. 4, after 72 and 96 h of exposure, the NS/PC proliferation rates in the sACM group, control shRNA sACM group and HMGB1 group increased significantly compared to the vehicle control group. Compared to the sACM group, the NS/PC proliferation rates in the HMGB1 shRNA sACM group exhibited a statistically significant decrease at 72 and 96 h, while the NS/PC proliferation rates in the control group showed no statistical differences at 72 or 96 h compared to the vehicle group (P>0.05).

### HMGB1 released by astrocytes promotes NS/PC proliferation in vitro by regulating the cell cycle

**Figure 5:**
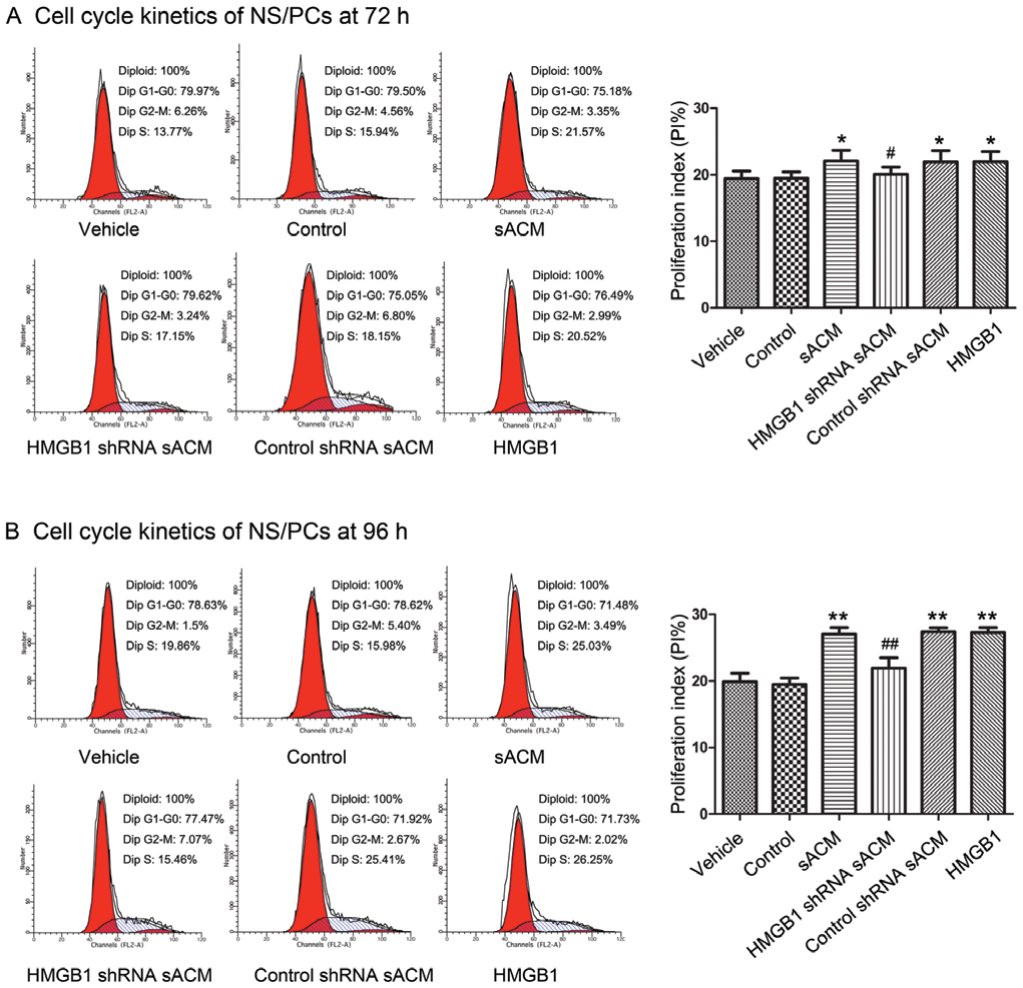
Astrocyte-derived HMGB1 on NS/PC proliferation was measured by analyzing the cell cycle. The cell cycle kinetics of NS/PCs were analyzed by flow cytometry at (A) 72 h and (B) 96 h. The proliferative capacity of NS/PCs was described as the proliferation index (PI): PI = (S + G2/M)/(G0/G1 + S + G2/M). The PIs of the sACM group, control shRNA sACM group and HMGB1 group were increased after 72 and 96 h. Compared with the sACM group, the PI of the HMGB1 shRNA sACM group was significantly decreased after 72 and 96 h. There were no intra-time point differences in PI between vehicle (NS/PC culture medium only) and control (normal ACM with IL-1β) groups at either the 72 or 96-h time point. ^*^P<0.01, ^**^P<0.001 vs. vehicle group; ^#^P<0.05, ^##^P<0.001 vs. sACM group.

To further investigate the effects of astrocyte-derived HMGB1 on NS/PC proliferation, we analyzed the cell cycle of NS/PCs by flow cytometry in a time course experiment using the different culture media. The PI was selected to reflect the proliferative ability of the NS/PCs, using the following formula: PI = (S + G2/M)/(G0/G1 + S + G2/M), as previously described (28). The PI in the NS/PC proliferation experiments is shown in Table III. There was no statistically significant difference in the PI following culture for 24 or 48 h (P>0.05) with the different culture media. Compared with the PI of the vehicle group, the PI of the sACM group, control shRNA sACM group and HMGB1 group showed a significant increase after 72 and 96 h (Fig. 5). Compared with the sACM group, the PI of the HMGB1 shRNA sACM group exhibited a statistically significant decrease after 72 and 96 h (Fig. 5). There was no significant difference in the PI between the vehicle group and the control group at all 4 time points (P>0.05).

### HMGB1 promotes NS/PC proliferation through the activation of the RAGE-dependent JNK signaling pathway

**Figure 6:**
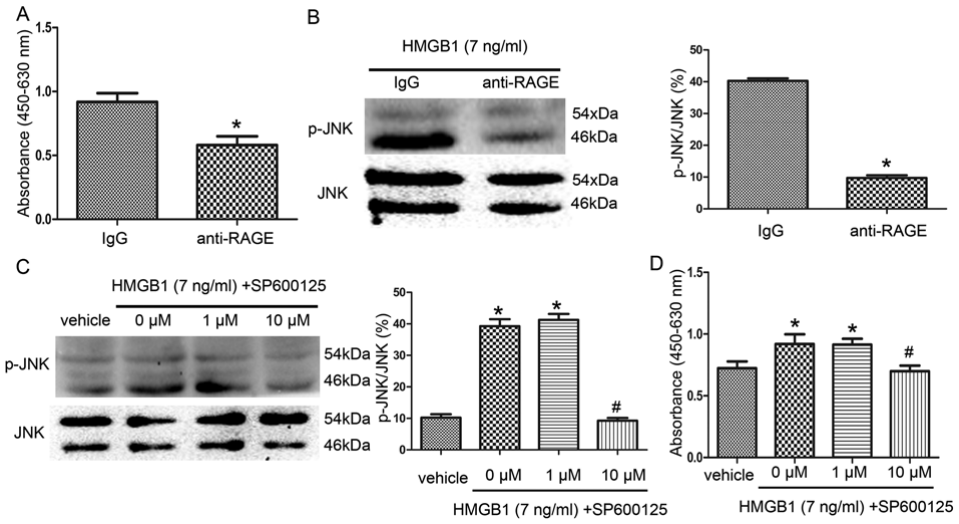
Analysis of signaling pathways involved in HMGB1-mediated NS/PC proliferation by CCK-8 assay (A and D) and western blot analysis (B and C). NS/PCs were cultured for 96 h in NS/PC culture medium containing 7 ng/ml HMGB1. (A) Compared to an IgG control, blockade of RAGE with an anti-RAGE antibody significantly inhibited HMGB1-induced NS/PC proliferation. ^*^P<0.001. (B) Western blot analysis showed that the blockade of RAGE reduced p-JNK levels in NS/PCs. ^*^P<0.001. (C) Exposure of NS/PCs to HMGB1-containing culture medium rapidly increased p-JNK levels in NS/PCs; the JNK inhibitor, SP600125, reduced JNK phosphorylation in the NS/PCs. ^*^P<0.001 vs. vehicle group; ^#^P<0.001 vs. 0 μM group. (D) SP600125 (10 μM) significantly reduced the ability of HMGB1 to enhance NS/PC proliferation. ^*^P<0.001 vs. vehicle group; ^#^P<0.001 vs. 0 μM group.

To explore the mechanisms behind HMGB1-mediated NS/PC proliferation, we investigated the RAGE receptor pathway, a critical receptor for HMGB1, and the JNK signaling pathway, a signaling pathway involved in HMGB1-RAGE signaling (30). First, we performed an *in vitro* CCK-8 assay in which the NS/PCs were placed in HMGB1 culture medium for 96 h with or without anti-RAGE antibodies. As shown in Fig. 6A, the NS/PC proliferation rates in the anti-RAGE group (absorbance, 0.581±0.068) decreased significantly compared with the IgG group (absorbance, 0.919±0.069). Correspondingly, the p-JNK protein levels were significantly attenuated in the anti-RAGE group compared to the IgG group (Fig. 6B).

To further determine the effects of the JNK signaling pathway on HMGB1-mediated NS/PC proliferation, the NS/PCs were incubated for 96 h in HMGB1 culture medium with various concentrations of the JNK inhibitor, SP600125. As shown in Fig. 6C, compared with the vehicle group, the p-JNK levels in the 0 μM group and 1 μM group were markedly increased, while the p-JNK levels significantly decreased in the 10 μM group compared to the 0 μM group. Correspondingly, the NS/PC proliferation rates significantly increased in the 0 μM group (absorbance, 0.920±0.078) and 1 μM group (absorbance, 0.914±0.047), compared with the vehicle group (absorbance, 0.723±0.055) (Fig. 6D), and the NS/PC proliferation rates were significantly decreased in the 10 μM group (absorbance, 0.700±0.045) relative to the 0 μM group.

## Discussion

In the present study, the CCK-8 proliferation assay and PI were selected to reflect the proliferative capacity of the NS/PCs. NS/PCs treated with IL-1β-stimulated ACM (sACM) displayed increased proliferation rates *in vitro*. When endogenous HMGB1 in the IL-1β-stimulated ACM was suppressed using shRNA, the ability of the sACM to evoke *in vitro* NS/PC proliferation was decreased. The addition of exogenous HMGB1 to the NS/PC cultures also increased the NS/PC proliferation rates *in vitro*. Furthermore, treatment of the HMGB1-induced NS/PCs with an anti-RAGE antibody significantly decreased both the NS/PC proliferation rates and p-JNK protein levels. Finally, treatment of the HMGB1-induced NS/PCs with various concentrations of the JNK inhibitor, SP600125, revealed that p-JNK levels decreased concomitantly with the proliferation rates of NS/PCs. These results suggest that astrocyte-derived HMGB1 promotes NS/PC proliferation *in vitro*, and that HMGB1-stimulated NS/PC proliferation may be mediated through the RAGE-dependent JNK signaling pathway.

As major components of the neurogenic niche, astrocytes play a crucial role in regulating neural stem cell proliferation and differentiation (1–4). However, following injury to the central nervous system, traditional thinking presumes that astrocytes are activated to form glial scars and promote inflammation, which is detrimental to neuronal recovery (31–34). However, emerging data suggest that the role of reactive astrocytes may be more complex than previously appreciated. It has been reported that reactive astrocytes expressing HMGB1 in the peri-infarct cortex may promote neurovascular remodeling and functional recovery after stroke, suggesting that these cells may in fact also possess beneficial effects (7,19). In the present study, we found that reactive ACM containing low levels of HMGB1 stimulated NS/PC proliferation *in vitro*. HMGB1 is a non-histone DNA-binding protein which stabilizes nucleosome formation and regulates gene expression (13,14). HMGB1 is also a multifunctional molecule that can act as a vital pro-inflammatory cytokine to trigger inflammation by stimulating the secretion of pro-inflammatory cytokines and chemokines, including tumor necrosis factor-α (TNF-α), IL-2, IL-6 and other cytokines (13,14). Hence, studies have focused on blocking HMGB1 signaling in order to reduce damage following brain injury (15,16).

In contrast to these negative effects, a growing body of literature indicates that HMGB1 may also have the potential to promote tissue regeneration. It has been reported that following a tissue lesion, HMGB1 can stimulate certain populations of cells to proliferate, such as mesoangioblasts (vessel-associated stem cells), myocardial cells and EPCs (7,35,36). It has also been reported that low levels of exogenous HMGB1 (1–10 ng/ml) promote EPC and neuronal precursor cell (NPC) proliferation *in vitro* (7,29). Previous studies have suggested that stimulated astrocytes can produce and release HMGB1 into the extracellular medium (8,9), and the release of HMGB1 from astrocytes has been shown to increase EPC proliferation both *in vitro* and *in vivo* (7). In this context, HMGB1 may also provide a potential missing link between reactive astrocytes and NS/PC proliferation, and the results presented in this study are consistent with this hypothesis. Firstly, when the expression of astrocytic HMGB1 was inhibited using shRNA, the ability of the sACM to promote NS/PC proliferation was blocked. Secondly, when exogenous HMGB1 was added to the NS/PC culture medium, the HMGB1 culture medium also enhanced the NS/PC proliferation rates. Taken together, these results suggest that it was HMGB1 in the sACM that increased NS/PC proliferation *in vitro*.

These experiments identified that astrocytic HMGB1 had a relatively modest and slow effect on NS/PC proliferation *in vitro*. Previous studies have identified that concentrations of HMGB1 ranging from 1 to 10 ng/ml significantly increase neural progenitor cell proliferation *in vitro* (29). Hayakawa *et al* (7) used multiple doses of HMGB1 and showed biphasic effects on proliferation, where low levels of HMGB1 (1–10 ng/ml) increased the proliferation rates of EPCs, but higher concentrations (100–1,000 ng/ml) appeared to have no effect, suggesting that high concentrations of HMGB1 may be deleterious by promoting endothelial inflammation and inducing neurotoxicity. In the experiments presented in this study, the concentration of HMGB1 in the sACM (~7 ng/ml) was within the limits of low levels of HMGB1 (1–10 ng/ml). We further identified that low levels of endogenous HMGB1 secreted from astrocytes can also increase NS/PC proliferation *in vitro*. In our experiments, the NS/PC proliferation rates exhibited a relatively stationary phase for up to 48 h and a statistically significant increased rate from 72 to 96 h. The time scales are consistent with previous findings reported by Meneghini *et al* (29) that HMGB1 produced a relatively stationary phase up to 48 h and a statistically significant increase at 72 h. In our experiments, we stimulated astrocytes with low levels of IL-1β *in vitro* to mimic the *in vivo* inflammatory microenviroment. Since others have reported that as little as 0.8 ng/ml IL-1β inhibits the proliferation of NPCs (37), and that astrocytes may release certain cytokines that affect NPC proliferation, we used a control group in which nACM was supplemented with IL-1β (0.1 ng/ml). We found that there was no effect on the proliferation of NS/PCs. In these experiments, we used ACM, but not NS/PC-astrocyte co-cultures; therefore, it is clear that molecules secreted by astrocytes, but not cell surface molecules on astrocytes, regulate NS/PC proliferation. All the findings in these *in vitro* experiments demonstrate that HMGB1 released from reactive astrocytes promotes the proliferation of NS/PCs.

We also provided evidence of the presence of a functional RAGE-JNK axis in HMGB1-mediated NS/PC proliferation. As a receptor of HMGB1, RAGE plays an important role in regulating stem cell proliferation and differentiation. Meneghini *et al* (29) reported that HMGB1 interacted with RAGE and stimulated both proliferation and neuronal differentiation of subventricular zone (SVZ)-derived NS/PCs *in vitro*. Additionally, Kim *et al* (38) demonstrated that HMGB1 may interact with RAGE and have important functions during the neuronal differentiation of NT2/D1 cells. In our *in vitro* NS/PC culture system, after blocking the activation of RAGE with a neutralizing antibody on the NS/PC surface, the ability of HMGB1 to enhance NS/PC proliferation was prevented. These findings suggest that RAGE also has an important function in the process of HMGB1-mediated NS/PC proliferation. Taguchi *et al* (30) reported that blocking RAGE-HMGB1-mediated cellular stimulation decreased the levels of p-JNK, and further inhibited the proliferation of rat C6 glioma cells. In our experiments, blocking RAGE with an anti-RAGE antibody in HMGB1 cultures also decreased p-JNK. To further demonstrate that the JNK signaling pathway is involved in HMGB1-induced NS/PC proliferation, we used the potent JNK inhibitor, SP600125. We found that HMGB1 culture medium increased p-JNK levels and the rate of NS/PC proliferation. When the p-JNK levels in the NS/PCs were reduced by SP600125, the HMGB1 cultured NC/PC proliferation rates decreased correspondingly. All these results indicated that after binding to RAGE, HMGB1 increased NS/PC proliferation by promoting JNK phosphorylation.

Nevertheless, the present study also has certain limitations. Firstly, in these experiments, we stimulated astrocytes with low levels of IL-1β *in vitro* to mimic an *in vivo* inflammatory microenviroment, and confirmed that HMGB1 released from astrocytes promoted NS/PC proliferation *in vitro*. However, the actual inflammatory microenviroment is far more intricate, and whether this mechanism also exists *in vivo* requires further investigation. Secondly, astrocytes may also release other cytokines, such as nerve growth factor, neurotrophic factor, ciliary neurotrophic factor and vascular endothelial growth factor (39–42). The elucidation of the mechanisms through which HMGB1 interacts with these other networks and pathways requires further investigation. Thirdly, there is no doubt that the mechanism through which HMGB1 promotes NS/PC proliferation is very complex, and whether other signaling pathways, such as the ERK and p38 MAPK signaling pathways (43) are also involved in this process requires more detailed investigation in future studies.

In conclusion, our study provides evidence that HMGB1 released from reactive astrocytes is sufficient to promote NS/PC proliferation, and the mechanisms through which HMGB1 promotes NS/PC proliferation may involve the activation of the RAGE-dependent JNK signaling pathway. Since the NS/PCs afford the plasticity to generate, repair and change nervous system function (44), the proliferation of NS/PCs may have beneficial effects in repairing brain damage. Our collective results support a previously described mechanism of a crosstalk between astrocytes and NS/PCs, and provide an experimental basis that reactive astrocyte-derived HMGB1 can promote NS/PC proliferation, which may be one of the reasons that NS/PCs spontaneously proliferate following brain injuries, and suggest that HMGB1 may be a very important factor for the regeneration of injured brain tissue. Further studies are required to investigate whether astrocyte-derived HMGB1 can induce NS/PCs to differentiate into neurons and to promote functional recovery following brain injury.

## Figures and Tables

**Table I:** Study design.

Experiment	Group	Number	Explanation
Astrocyte-conditioned media (ACM) synthesis of HMGB1	nACM	9	Astrocytes were cultured in NS/PC medium for 24 h in the absence of IL-1β stimulation
	sACM	9	Astrocytes were cultured for 24 h in NS/PC medium containing IL-1β (0.1 ng/ml; Prospec, East Brunswick, NJ, USA)
	HMGB1 shRNA sACM	9	Astrocytes expressing HMGB1 shRNA were cultured for 24 h in NS/PC medium containing 0.1 ng/ml IL-1β
	Control shRNA sACM	9	Astrocytes expressing control shRNA were cultured for 24 h in NS/PCs medium containing 0.1 ng/ml IL-1β
NS/PC proliferation	Vehicle	9^a^	NS/PCs were cultured in serum-free NS/PC medium
	Control	9^a^	NS/PCs were cultured in nACM containing IL-1β
	sACM	9^a^	NS/PCs were cultured in sACM
	HMGB1 shRNA sACM	9^a^	NS/PCs were cultured in sACM from astrocytes expressing HMGB1 shRNA
	Control shRNA sACM	9^a^	NS/PCs were cultured in sACM from astrocytes expressing control shRNA
	HMGB1	9^a^	NS/PCs were cultured in serum-free NS/PC medium containing 7 ng/ml recombinant human HMGB1 (Prospec)
RAGE pathway experiment	IgG	9	NS/PCs were cultured for 96 h in NS/PC culture medium containing 7 ng/ml HMGB1 and 20 μg/ml control IgG (Beyotime Institute of Biotechnology, Wuhan, China)
	Anti-RAGE	9	NS/PCs were cultured for 96 h in NS/PC culture medium containing 7 ng/ml HMGB1 and 20 μg/ml anti-RAGE antibody (Santa Cruz Biotechnology, Santa Cruz, CA, USA)
JNK pathway analysis	Vehicle	9	NS/PCs were cultured for 96 h in serum-free NS/PC medium
	0 μM	9	NS/PCs were cultured for 96 h in NS/PC culture medium containing 7 ng/ml HMGB1
	1 μM	9	NS/PCs were cultured for 96 h in NS/PC culture medium containing 7 ng/ml HMGB1 and 1 μM SP600125
	10 μM	9	NS/PCs were cultured for 96 h in NS/PC culture medium containing 7 ng/ml HMGB1 and 10 μM SP600125

a Indicates 9 samples at each time point (24, 48, 72 and 96 h).

**Table II:** Effects of astrocyte-conditioned medium on the CCK-8 absorbance of NS/PCs (n=9, values indicate the means ± SD).

Time point (h)	Vehicle	Control	sACM	sACM HMGB1 shRNA	sACM control shRNA	HMGB1
24	0.197±0.036	0.196±0.033	0.175±0.027	0.196±0.032	0.179±0.026	0.180±0.033
48	0.294±0.041	0.298±0.048	0.319±0.050	0.313±0.039	0.310±0.046	0.312±0.031
72	0.404±0.047	0.413±0.049	0.490±0.041	0.423±0.030	0.487±0.041	0.479±0.045
96	0.765±0.059	0.745±0.071	0.966±0.056	0.848±0.051	0.980±0.053	0.984±0.073

**Table III:** Effects of astrocyte-conditioned medium on the proliferation index (PI) of NS/PCs (n=9, values indicate the means ± SD).

Time point (h)	Vehicle	Control	sACM	sACM HMGB1 shRNA	sACM control shRNA	HMGB1
24 h	17.87±0.92	17.87±0.86	17.69±0.97	17.72±0.96	17.84±1.09	17.59±1.02
48 h	18.95±0.96	18.58±1.00	18.81±0.96	19.09±1.07	18.65±1.07	18.58±0.92
72 h	19.45±1.12	19.48±0.97	22.07±1.61	20.06±1.00	21.95±1.69	21.96±1.53
96 h	19.89±1.27	19.48±0.96	27.04±1.00	21.13±1.09	27.38±0.63	27.82±0.76

PI = (S + G2/M)/(G0/G1 + S + G2/M).
